# Efficiency enhancements in Ag nanoparticles-SiO_2_-TiO_2_ sandwiched structure via plasmonic effect-enhanced light capturing

**DOI:** 10.1186/1556-276X-8-73

**Published:** 2013-02-12

**Authors:** Jinxia Xu, Xiangheng Xiao, Andrey L Stepanov, Fen Ren, Wei Wu, Guangxu Cai, Shaofeng Zhang, Zhigao Dai, Fei Mei, Changzhong Jiang

**Affiliations:** 1 Department of Physics and Key Laboratory of Artificial Micro- and Nano-structures of Ministry of Education, Wuhan University, Wuhan, 430072, People’s Republic of China; 2 Center for Electron Microscopy and Hubei Nuclear Solid Physics Key Laboratory, Wuhan University, Wuhan, 430072, People’s Republic of China; 3 Kazan Physical-Technical Institute, Russian Academy of Sciences, Kazan, 420029, Russia; 4 School of Electrical and Electronic Engineering, Hubei University of Technology, Wuhan, 430068, People’s Republic of China

**Keywords:** Plasmonic effect, Ion implantation, Ag nanoparticles, Photocatalysis

## Abstract

TiO_2_-SiO_2_-Ag composites are fabricated by depositing TiO_2_ films on silica substrates embedded with Ag nanoparticles. Enhancement of light absorption of the nanostructural composites is observed. The light absorption enhancement of the synthesized structure in comparison to TiO_2_ originated from the near-field enhancement caused by the plasmonic effect of Ag nanoparticles, which can be demonstrated by the optical absorption spectra, Raman scattering investigation, and the increase of the photocatalytic activity. The embedded Ag nanoparticles are formed by ion implantation, which effectively prevents Ag to be oxidized through direct contact with TiO_2_. The suggested incorporation of plasmonic nanostructures shows a great potential application in a highly efficient photocatalyst and ultra-thin solar cell.

## Background

Titanium dioxide (TiO_2_) has strong photocatalytic activity, high chemical stability, a long lifetime of photon-generated carriers, nontoxicity, and low cost, which make it one of the most widely used photocatalysts for hydrogen production and solar cells, as well as water and air remediation [1-3]. At modern times, TiO_2_ becomes a hot research topic because of the potential applications in the field of environment and energy [4-6]. Unfortunately, owing to its wide band gap of 3.2 eV (at 390 nm), only approximately 4% solar spectrum can be utilized. During the last decades, great efforts have been made to modify the TiO_2_ to enhance the visible light response. A considerable increase in the photocatalytic activity in the visible region has been observed by doping [7-10]. However, to date, the doping structure lacks reliable controllability. Recently, metallic nanostructures have been introduced into a semiconductor film (e.g., ZnO, InGaN quantum wells) for enhancement of light emission, photocurrent solar cells [11-14], and photocatalysts [15-17] by a strong plasmonic effect of metallic nanostructures. In order to maximize the utilization rate of the UV region of the sunlight, in this letter, we design a new composite structure to enhance the light absorption efficiency by coupling TiO_2_ to Ag nanoparticles (NPs) embedded in SiO_2_ formed by low-energy Ag ion implantation. Ag NPs show a very intense localized surface plasmon resonance (SPR) in the near-UV region [18], which strongly enhances the electric field in the vicinity of the Ag NPs. This enhanced electric field at the near-UV region could increase the UV light absorption to boost the excitation of electron–hole pairs in TiO_2_ and thus increase the photoelectric conversion efficiency. In this kind of structure, the Ag NPs embedded in SiO_2_ serve two purposes. Firstly, SiO_2_ as a protective layer prevents Ag to be oxidized through direct contact with TiO_2_. Secondly, the size and depth distributions of the embedded Ag NPs can be controlled by choosing implantation parameters and post-implantation thermal treatment [19], which can tune the SPR spectrum of Ag NPs to match the absorption edge of TiO_2_. Thus, it is possible to design nanostructures that concentrate the light surrounding near Ag NPs, which enhance the light absorption of the TiO_2_ film.

## Methods

High-purity silica slides were implanted by Ag ions at 20, 40, and 60 kV to a fluence of 5 × 10^16^ ions/cm^2^ and at 40 kV to 1 × 10^17^ ions/cm^2^ using a metal vapor vacuum arc ion source implanter, respectively. The TiO_2_-SiO_2_-Ag nanostructural composites were obtained by depositing TiO_2_ films (100 nm thick) on the surface of the as-implanted silica substrates using a direct-current reactive magnetron sputtering system. For comparison, an un-implanted silica substrate was deposited with the TiO_2_ film under the same growth condition. Subsequently, all deposited samples were annealed at 500°C in oxygen gas for 2 h to obtain an anatase-phase TiO_2_ film. The TiO_2_-covered silica substrates with embedded Ag NPs are named S1 to S4 as shown in Table 1. The optical absorption spectra of all the samples were measured using a UV–vis-NIR dual-beam spectrometer (Shimadzu UV 2550, Shimadzu Corporation, Kyoto, Japan) with wavelengths varying from 200 to 800 nm. Raman scattering spectra of all the samples were collected using a micro-Raman system (LabRAM HR800, HORIBA Jobin Yvon Inc., Edison, NJ, USA). An Ar laser (488.0 nm) was used as the excitation source, and the laser power was kept at 10 mW. The microstructure of the samples was investigated using JEOL JEM 2010 (HT) transmission electron microscopes (TEM; JEOL Ltd., Tokyo, Japan) operated at 200 kV.

**Table 1:** Ag ion implantation parameters for all samples

**Sample**	**Fluence of ion implantation (ions/cm**^**2**^**)**	**Energy of ion implantation (kV)**
S1	5 × 10^16^	20
S2	5 × 10^16^	40
S3	1 × 10^17^	40
S4	5 × 10^16^	60

The photocatalytic efficiencies of TiO_2_ and TiO_2_-SiO_2_-Ag nanostructural composites with an area of 4 cm^2^ were evaluated by measuring the degradation rates of 5 mg/L methylene blue (MB) solution under UV–vis irradiation. A mercury lamp (Osram 250 W (Osram GmbH, Munich, Germany) with a characteristic wavelength at 365 nm) was used as a light source. The TiO_2_ and the TiO_2_-SiO_2_-Ag composite films were placed in 40 mL of MB solution with a concentration of 5 mg/L. Before irradiation, the samples were put in 40 mL of MB solution for 30 min in the darkness to reach absorption equilibrium. The decolorization of the MB solution was measured by an UV–vis spectrometer (Shimadzu UV 2550, Shimadzu Corporation) at the wavelength of 664.0 nm. The absorption spectrum of the MB solution was measured at a time interval of 30 min, and the total irradiation time was 4 h.

## Results and discussion

**Figure 1:**
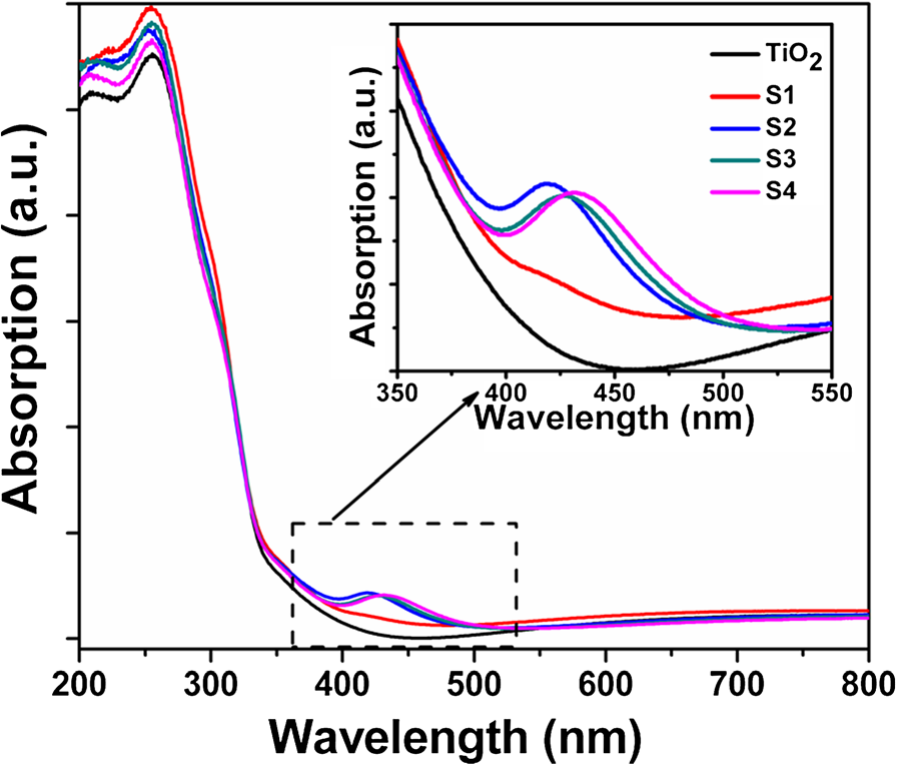
**The optical absorption spectra of S1 to S4 and the pure TiO**_**2 **_**film.**

Figure 1 shows the optical absorption spectra of S1 to S4 and the TiO_2_ films. The absorption edge around 390 nm belongs to the intrinsic exciton absorption of TiO_2_[20]. The obvious absorption peaks at about 419 to 433 nm can be attributed to the SPR of Ag NPs formed by Ag ion implantation [21]. As seen, the SPR of Ag NPs is close to the exciton edge (around 390 nm) of anatase TiO_2_. Therefore, it is expected that an efficient energy transfer from the Ag NPs to TiO_2_ can occur. The position of the Ag SPR absorption peak of S2 is around 419 nm, which is a blue shift compared to that of the other three samples. The SPR peak of S2 is closest to the anatase TiO_2_ exciton energy; therefore, the strongest resonant coupling effect between Ag SPR and the excitons of the TiO_2_ films may be produced more effectively.

**Figure 2:**
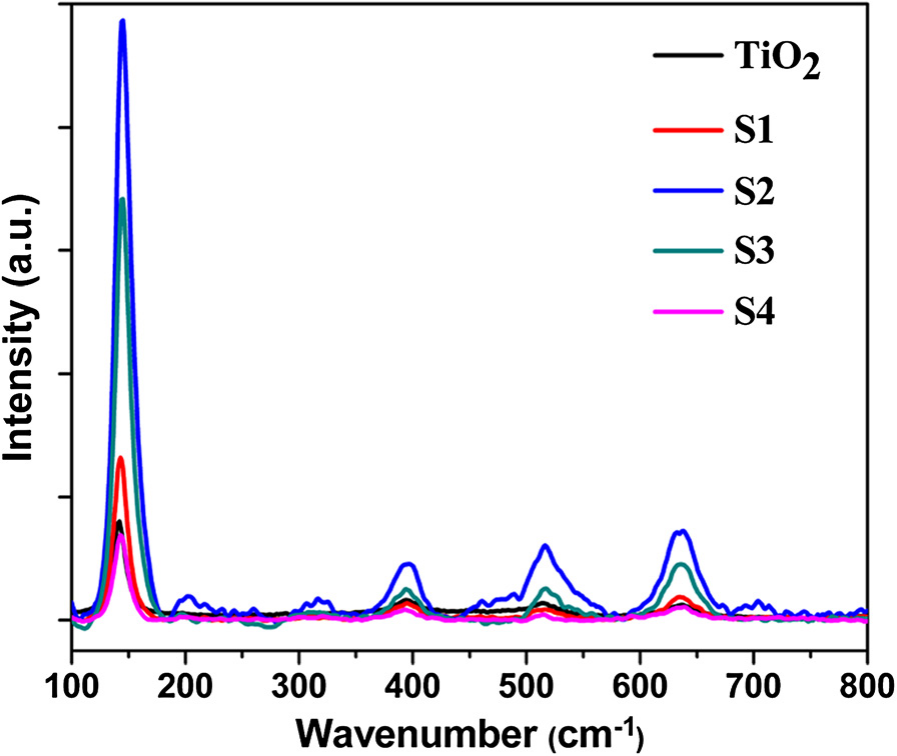
**The Raman scattering spectra of S1 to S4 and the pure TiO**_**2 **_**film.**

To illustrate the strong near field induced by the SPR of Ag NPs, the Raman scattering spectra of S1 to S4 and TiO_2_ are measured as presented in Figure 2. The observed Raman bands at 144, 199, 399, 516, and 640 cm^−1^ can be assigned to the Eg, Eg, B1g, A1g, or B1g and Eg vibration modes of anatase phase, respectively, which are consistent with the characteristic patterns of pure anatase without any trace of a rutile or brookite phase [22]. It is found that the Raman intensity for S1 to S4 increases compared to that of TiO_2_, and S2 shows the strongest Raman intensity. It is well known that Raman scattering intensity is proportional to the square of the electric field intensity [23]; thus, stronger Raman scattering attained from the TiO_2_-SiO_2_-Ag structure indicates that a stronger electric field is induced by Ag NPs embedded in the SiO_2_ substrate. When the Ag NPs are irradiated by a laser in the spectral area of the particle absorption band’s longer wavelength shoulder, a strong near field is produced due to the SPR, so Raman scattering is enhanced. As seen from Figure 2, the enhancement factors of Raman scattering of S1 to S4 are different because of various coupling field efficiencies. Thus, it is possible to conclude that the implantation energy and fluence have determined the Raman scattering enhancement factor.

**Figure 3:**
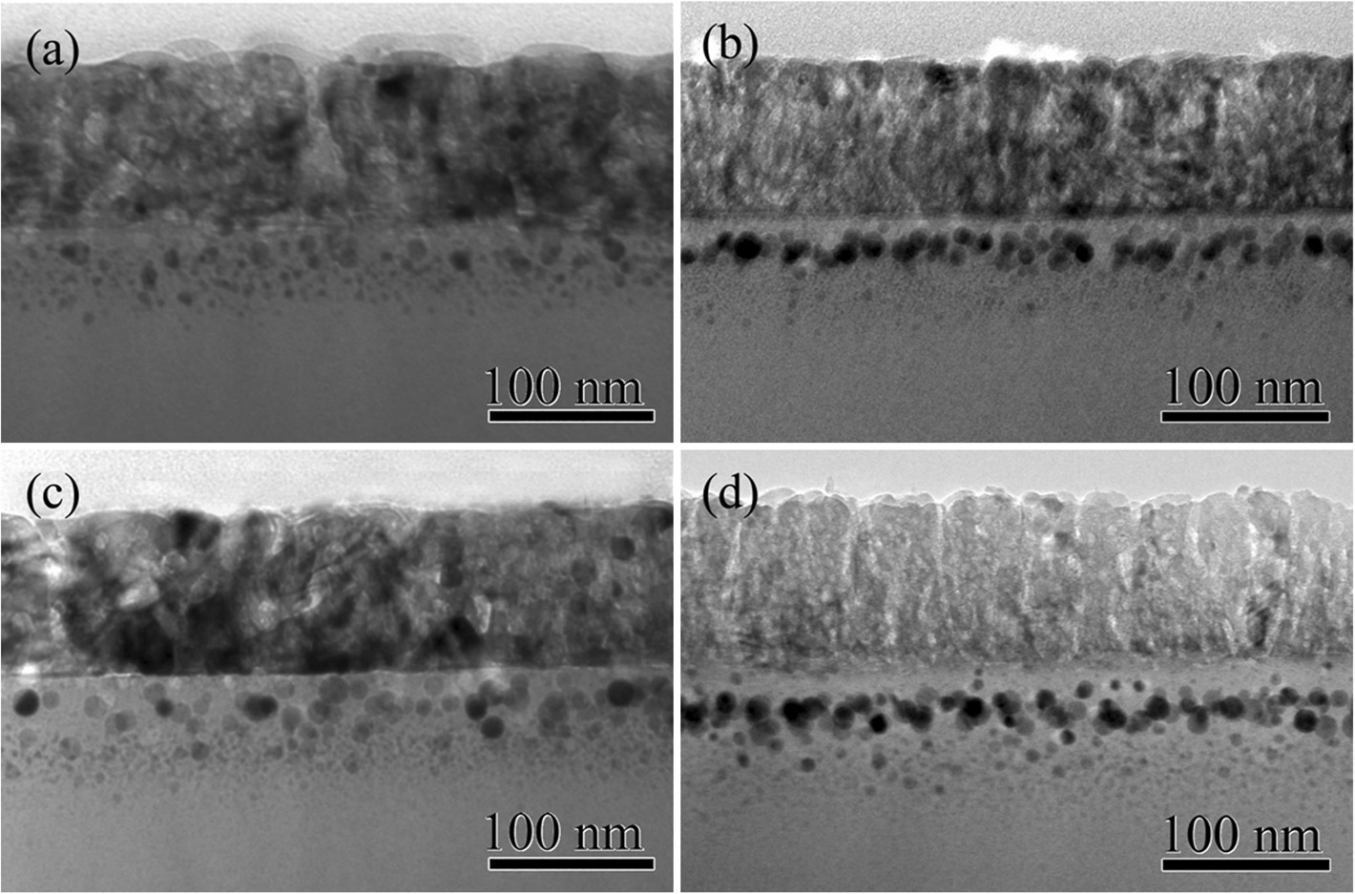
Cross-sectional TEM images of (a) S1, (b) S2, (c) S3, and (d) S4.

To understand the relationship between the size and depth distributions of the Ag NPs in silica glass and the Raman scattering enhancement factor of the TiO_2_-SiO_2_-Ag nanocomposites, the microstructural characterization of S1 to S4 was investigated by TEM as shown in Figure 3. The TEM image of S1 (Figure 3a) shows that the size of the Ag NPs appears to have a wide distribution. However, increasing the implantation energy to 40 kV as shown in Figure 3b, the Ag NPs in S2 are quite uniform in size (with a size of 20 nm) and distribute at nearly the same depth of 7 nm from the surface. Under high energy ion implantation, more heat will be induced in the sample in a short time, which enhances the diffusion of Ag atoms. Therefore, the implanted Ag ions trend to aggregate to larger NPs around the projected range [24-26]. The near field induced by the SPR of the Ag NPs is very strong due to the presence of the formed Ag NPs with bigger size and the near-field dipolar interactions between adjacent particles [27]. On the other hand, the dipolar interactions between adjacent particles with nearly the same size can result in a blue shift of SPR [28]; thus, the blue shift in the SPR peak of the Ag NPs is observed in Figure 1, which may produce a strongest resonant coupling effect between the SPR of Ag NPs and TiO_2_. It means that the stronger near field can be induced. In this case, S2 has the strongest Raman scattering enhancement factor. The size of the Ag NPs in S1 is smaller, and the distribution is wider than that in S2. It means that the near field induced by SPR of the Ag NPs in S1 is weaker than that in S2. Further increasing the implantation energy to 60 kV as presented in Figure 3d, the Ag NPs in S4 reside deeper below the surface than those in S2. Since the SP is an evanescent wave that exponentially decays with distance from the metal particles to the surface [29], the enhancement of Raman scattering decreases progressively with the increase of distance between the Ag NPs with the TiO_2_ film; therefore, Raman scattering intensity of S4 has almost no enhancement. When the ion implantation fluence is increased to 1 × 10^17^ ions/cm^2^ with an implantation energy of 40 kV (S3) as displayed in Figure 3c, large Ag NPs with a size of about 15 nm are formed near the surface and the small ones in the deeper SiO_2_ matrix. The surface sputtering effect plays an important role for ion implantation at high fluence. The formed small Ag NPs near the surface are sputtered away by the subsequent implanted ions; as a result, the large Ag NPs are populated near the surface of S3 [24]. The Raman scattering enhancement factor is small with increasing implantation fluence. Therefore, the Raman scattering enhancement demonstrates that the strong near field is actually induced by introducing Ag NPs. The increased field could locally concentrate the light surrounding the Ag NPs and thus enhance the absorption of light.

**Figure 4:**
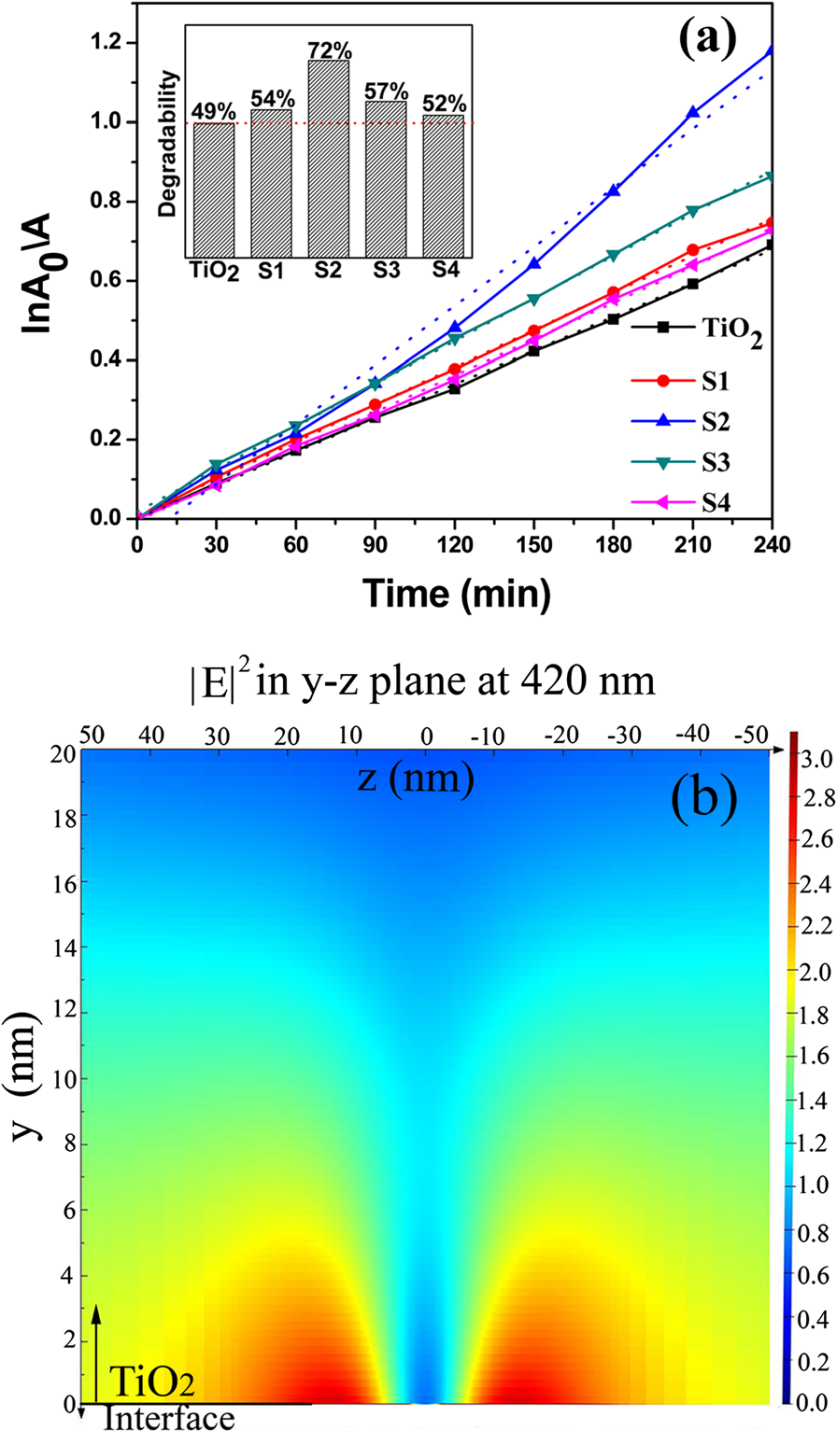
**Photodegradation of MB and amplitude enhancement of electric field.** (**a**) The photodegradation of MB solution by S1 to S4 and reference sample TiO_2_ under UV light irradiation (inset) and the corresponding plots of $\mathrm{In}\left(\frac{A_{0}}{A}\right)$ versus the irradiation time, showing the linear fitting results. (**b**) Amplitude enhancement of the electric field inside a TiO_2_ layer is simulated by the FDTD method.

In order to study the enhancement of light absorption in TiO_2_-SiO_2_-Ag nanostructural composites, the photocatalytic activities of S1 to S4 are investigated by the UV degradation of the MB solution at room temperature. For comparison, the TiO_2_ film is carried out under the same experimental conditions. As shown in Figure 4a (inset), the concentration of MB is decreased upon the irradiation time, and the TiO_2_ film can decompose 49% of MB after the UV irradiation for 4 h. However, the TiO_2_-SiO_2_-Ag nanostructural composite films obtained a higher photocatalytic efficiency than the pure TiO_2_ film, and S2 has the highest photocatalytic efficiency compared to the other three samples and degraded 72% of MB. The enhancement ratio is as high as 47%. Meanwhile, the photodegradation of MB can be assumed to follow the classical Langmuir-Hinshelwood kinetics [30], and its kinetics can be expressed as follows:

(1) $$\mathrm{In}\left(\frac{A_{0}}{A}\right)=\mathrm{kt}\text{,}$$

where *k* is the apparent first-order reaction rate constant (min^−1^), and *A*_0_ and *A* represent the absorbance before and after irradiation for time *t*, respectively. As displayed in Figure 4a, S2 shows the highest rate constant among all the samples. The *k* values of S2 are about two times than those of pure TiO_2_. The kinetic rate constants follow the order S2 > S3 > S1 > S4 > TiO_2_. This is consistent with the Raman scattering enhancement result.

The near-field enhancement in the TiO_2_ layer due to the presence of the Ag NPs is also simulated using the finite-difference time-domain (FDTD) method as shown in Figure 4b. In our structure, we consider *x* as the light incident direction, the illuminating plane wave with a wavelength of 420 nm is *y* polarized, an Ag NP with a diameter of 20 nm is embedded in SiO_2_, and the distance to the surface of the SiO_2_ substrate is 7 nm. An amplitude enhancement to 3 can be observed. Theoretical and experimental results show that an enhancement of the near field is induced by the SPR of Ag NPs. The SPR excitations cause a large increase in electromagnetic field in the vicinity of metal NPs. The localized amplification can increase the incident excitation field and boost the creation of hole–electron pairs, which results in the enhancement of the photocatalytic activity of TiO_2_.

## Conclusions

In conclusion, we have successfully demonstrated a plasmonic effect by simply incorporating Ag NPs with TiO_2_ film. Optimum ion implantation conditions for Ag NPs synthesis in SiO_2_ were experimentally estimated. The plasmonic effect occurring near the interface of TiO_2_ and silica glass has effectively enhanced the light trapping. Both the experimental data and the simulations show that the enhancement effect is attained from the near-field enhancement induced by the SPR of Ag NPs. Our results have shown that the plasmonic effect has great potential in the application of increasing the UV light absorption in TiO_2_ photocatalysts and opening up opportunities for highly efficient ultra-thin film solar cells.

## Competing interests

The authors declare that they have no competing interests.

## Authors’ contributions

JX participated in the material preparation and data analysis and drafted the manuscript. XX conceived and co-wrote the paper. AS, FR, WW, GC, SZ, ZD, and FM participated in the sample characterization. CJ participated in its design and coordination. All authors read and approved the final manuscript.
